# Characterization of *Treponema denticola* Mutants Defective in the Major Antigenic Proteins, Msp and TmpC

**DOI:** 10.1371/journal.pone.0113565

**Published:** 2014-11-17

**Authors:** Yuki Abiko, Keiji Nagano, Yasuo Yoshida, Fuminobu Yoshimura

**Affiliations:** Department of Microbiology, School of Dentistry, Aichi Gakuin University Nagoya, Aichi, Japan; University of North Dakota School of Medicine and Health Sciences, United States of America

## Abstract

*Treponema denticola*, a gram-negative and anaerobic spirochete, is associated with advancing severity of chronic periodontitis. In this study, we confirmed that two major antigenic proteinswere Msp and TmpC, and examined their physiological and pathological roles using gene-deletion mutants. Msp formed a large complex that localized to the outer membrane, while TmpC existed as a monomer and largely localized to the inner membrane. However, TmpC was also detected in the outer membrane fraction, but its cell-surface exposure was not detected. Msp defects increased cell-surface hydrophobicity and secretion of TNF-α from macrophage-like cells, whereas TmpC defects decreased autoagglutination and chymotrypsin-like protease activities. Both mutants adhered to gingival epithelial cells similarly to the wild-type and showed slightly decreased motility. In addition, in Msp-defective mutants, the TDE1072 protein, which is a major membrane protein, was abolished; therefore, phenotypic changes in the mutant can be, at least in part, attributed to the loss of the TDE1072 protein. Thus, the major antigenic proteins, Msp and TmpC, have significant and diverse impacts on the characteristics of *T*. *denticola*, especially cell surface properties.

## Introduction

Periodontitis is a chronic gingival inflammatory disease, which is caused by the presence of multi-species biofilms in the gingival crevice [1]. *Treponema denticola*, a gram-negative and anaerobic spirochete, is thought to be responsible for the advance of disease severity [2]. *T*. *denticola* possesses several characteristics, such as high motility, protease production, and an unusual surface layer (outer membrane), which are associated with pathogenicity [3], [4]. The bacteria, by using periplasmic flagella, are capable of motion even in highly viscous environments. *T*. *denticola* produces a large amount of a chymotrypsin-like protease (CTLP) known as dentilisin, which is a complex molecule consisting of PrcA, PrcB, and PrtP proteins [5]. The bacterium also produces a trypsin-like protease [6]. The outer membrane of *T*. *denticola* is unusual in that it is devoid of lipopolysaccharides (LPS), which are commonly found in gram-negative bacteria; instead, it contains a lipid that is similar to lipoteichoic acid, which is found in gram-positive bacteria [7].

In a previous study, we found that *T*. *denticola* possesses only a few antigenic proteins [8]. Briefly, an antiserum prepared by subcutaneously injecting *T*. *denticola* whole cells into a rabbit (anti-*T*. *denticola* antiserum) detected a few distinguishable bands in western blot analysis, using the bacterial cell lysate as an antigen. We confirmed that the two most intense bands were Msp and TmpC. Msp is a well-known major antigen in *T*. *denticola* [9]–[12]. Msp is abundantly expressed in the bacteria and is partially exposed on the cell surface [13]. Msp functions in adhesion to other bacteria [14] and host components [15]–[17]. Further, Msp was reported to be a porin [18], which acts as a permeable pore in the outer membrane of gram-negative bacteria and contributes to antibiotic resistance [19]. TmpC has also been reported to function as an antigenic protein in *T*. *denticola* [11]. Additionally, the antigenicity of TmpC was shown in the congeneric bacterium *T*. *pallidum* [20], [21]. In *T*. *pallidum*, TmpC was lipidated [20] and played a role as a purine nucleoside receptor [22]. However, to the best of our knowledge, the details of TmpC function in *T*. *denticola* remain unknown.

In the present study, we describe the major antigenic proteins detected in our previous study (unpublished data) [8]. We also report here on the physiological and pathological roles of the major antigenic proteins, Msp and TmpC, in *T*. *denticola,* which we investigated using mutants with defects in these proteins.

## Methods

### *T*. *denticola* strains and culture conditions

*T*. *denticola* ATCC 35405 (wild type, WT; RIKEN BioResource Center, Ibaraki, Japan) and isogenic mutants were anaerobically and statically cultivated in Modified GAM broth (Nissui Pharmaceutical Co., Ltd., Tokyo, Japan) [23] supplemented with 0.001% (w/v) thiamine pyrophosphate and 5% (v/v) heat-inactivated rabbit serum (mGAM-TS) at 37°C. Growth was monitored by measuring the OD_660_, and *T*. *denticola* cells in the early stationary phase were used for each experiment, unless otherwise noted. When needed, high-purity agar (Agar Noble, Becton, Dickinson and Company, Franklin Lakes, NJ, USA) and antibiotics (described in detail below) were added to the medium.

### Animal experiments

Animal experiments were performed in strict accordance with the recommendations of the Regulations on Animal Experimentation of Aichi Gakuin University. The protocols were approved by the Aichi Gakuin University Animal Research Committee (permit numbers: AGUD 254 and 255). All efforts were made to minimize animal suffering, and animals were killed under sodium pentobarbital anesthesia.

### Antibiotics and antibiotic sensitivity test

For the selection of transgenic mutants and antibiotic sensitivity testing, we used the following antibiotics: ampicillin, erythromycin, gentamicin, kanamycin, metronidazole, penicillin G, tetracycline, and vancomycin (all were obtained from Sigma-Aldrich Co., St. Louis, MO, USA). The minimum inhibitory concentration (MIC) was determined by employing a liquid dilution assay. Briefly, bacterial cultures were inoculated into mGAM-TS broth at an OD_620_ of 0.1. After 5 days of anaerobic incubation, the turbidity (OD_620_) was measured to determine whether or not the bacteria grew.

### Subcellular fractionation

The following procedures were performed under cold conditions. *T*. *denticola* cells were washed in a buffer consisting of 20 mM Tris/HCl, pH 7.5, supplemented with protease inhibitors (1 mM phenylmethylsulfonyl fluoride, 0.1 mM *N*α-*p*-tosyl-L-lysine chloromethyl ketone and 0.1 mM leupeptin). The cells were disrupted in a French pressure cell three passages at 7.3 MPa. Unbroken cells were removed by centrifugation at 1,000×*g* for 10 min. The resultant whole-cell lysate was subjected to ultracentrifugation at 100,000×*g* for 60 min. The supernatant and the sediment were collected as the soluble and envelope fractions, respectively. The envelope fraction was suspended in 20 mM Tris/HCl, pH 7.5 using a glass homogenizer. The protein concentration was measured using a Pierce BCA Protein Assay kit (Thermo Scientific, Rockford, IL, USA).

The surface layer (outer membrane) was extracted from intact cells of *T*. *denticola,* as described previously [8]. Briefly, bacterial cells were gently suspended in phosphate-buffered saline (PBS), pH 7.4, supplemented with 0.1% (w/v) Triton X-100 and protease inhibitors. The suspension was then centrifuged at 4,000×*g* for 15 min to separate the fraction containing the outer membrane from the whole cells. The supernatant was filtrated through a 0.22-µm pore filter membrane to remove residual cells. It should be noted that the supernatant fraction probably contained soluble molecules derived from the periplasmic space, in addition to the outer membrane. The remaining pelleted cell body (containing the inner membrane) was suspended in the same volume of PBS with protease inhibitors, then disrupted by sonication.

### Preparation of antiserum to TmpC

The *tmpC* gene, encoding the entire TmpC protein, was amplified by PCR from the chromosomal DNA of *T*. *denticola* ATCC 35405 using the primers His-tmpC-F and His-tmpC-R, to which restriction enzyme recognition sites had been appended (Table 1). The DNA fragments were temporarily cloned into the pGEM-T Easy vector (Promega Corporation, Madison, WI, USA) and sequenced to confirm their identity. The *tmpC* gene was transferred to the pET28(b) plasmid (Novagen, Darmstadt, Germany) for addition of a hexa-histidine (His) tag to the N-terminus of TmpC and then introduced into *Escherichia coli* BL21(DE3). The His-tagged TmpC was purified using a cobalt-affinity column, emulsified with complete Freund’s adjuvant, and injected into a rabbit to obtain anti-TmpC antiserum.

**Table 1:** Primers used in this study.

Primer	Sequence (5′ to 3′)	Description
His-tmpC-F	CTGACTTCTGCTAGCAAAGAAGAAGGCAAAAAG	Forward primer to amplify *tmpC*, inserted *Nhe*I-digestion site indicated by underline
His-tmpC-R	ACTTCTTTCTCGAGTTATTTAAATAAATCGCCTTG	Reverse primer to amplify *tmpC*, inserted *Xho*I-digestion site indicated by underline
tmpCU-F	AAATGAAGTAGTCTCCGCTGATGG	Forward primer to amplify upstream region of *tmpC*
tmpCU-R	GAAGGATGAAATTTTTCAGGGACAACTT AGAAACT CCTTTTTTGTATGTTAATTGCCAAAAAATTAAG	Reverse primer to amplify upstream region of *tmpC*, fused with upstream region of *ermB* indicated by underline
tmpCD-F	CTATGAGTCGCTTTTGTAAATTTGGAAAG AACTTAA AGAAGTTTACAAAGCCCTCTTTCTACAG	Forward primer to amplify downstream region of *tmpC*, fused with terminal region of *ermB* indicated by underline
tmpCD-R	GCATCAGGTTCAATAAAAAGACCGTATTTATC	Reverse primer to amplify downstream region of *tmpC*
ermB-F	AAGTTGTCCCTGAAAAATTTCATCCTTC	Forward primer to amplify *ermB*
ermB-R	CTTTCCAAATTTACAAAAGCGACTCATAG	Reverse primer to amplify *ermB*
1949-F	CGATGAACCTACGGCAGTTT	Forward primer to anneal within TDE1949, downstream gene of *tmpC*
1949-R	GACCGTGTTTTTGCCTGTC	Reverse primer to anneal within TDE1949
0406-F	GAATACCGCAATACCCTCAAAG	Forward primer to anneal within TDE0406, downstream gene of *msp*
0406-R	CATCATTCGGAA AGCCTA AGC	Reverse primer to anneal within TDE0406
1072-F	CAAGGCTCCCTATGTTTGGAAG	Forward primer to anneal within TDE1072
1072-R	CATAGTTGGCAGGGTTTCCG	Reverse primer to anneal within TDE1072

### SDS-PAGE and western blot analyses

**Figure 1:**
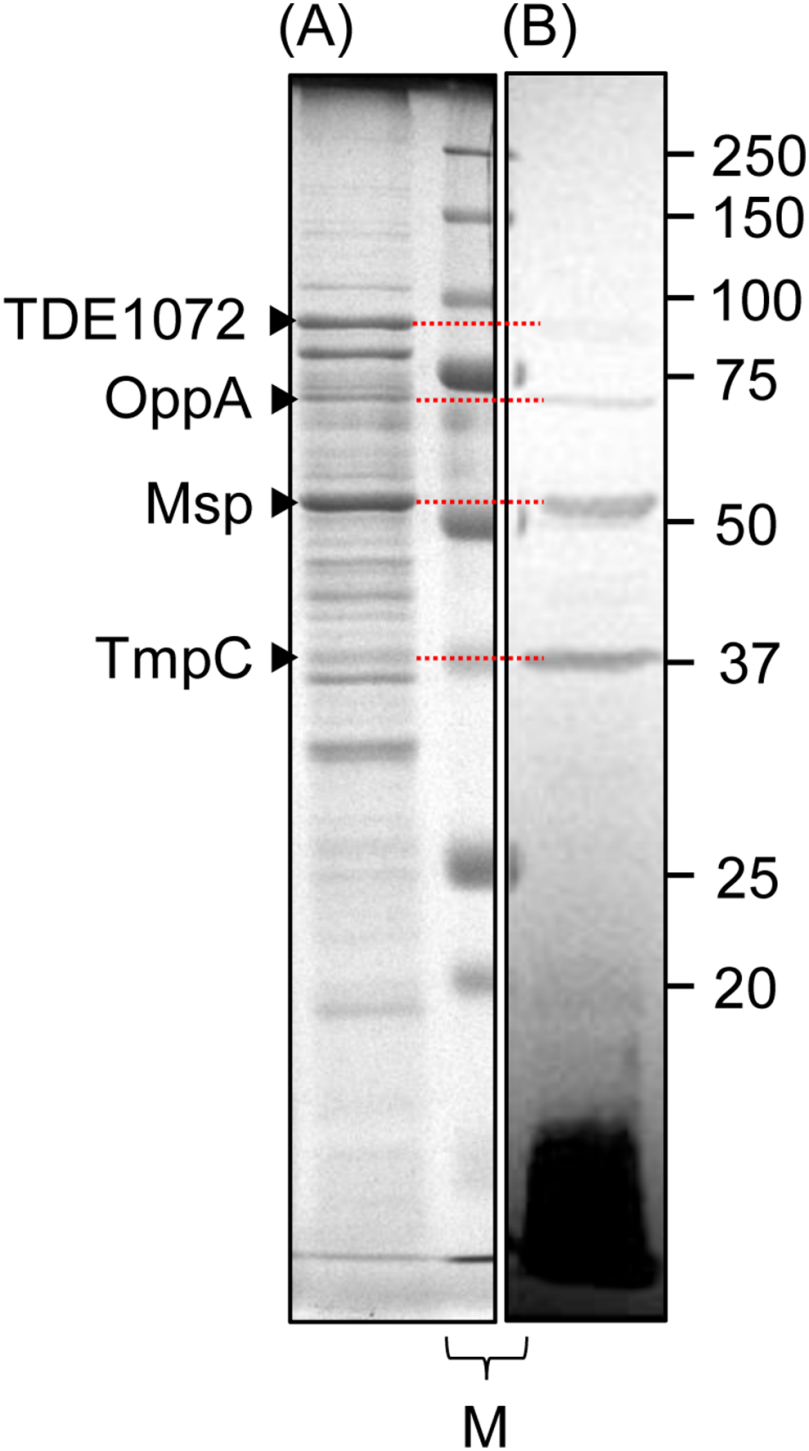
Identification of major antigenic proteins in *T*. *denticola*. The envelope fraction of the wild type of *T*. *denticola* was applied to two lanes across a molecular-weight standard in an SDS-PAGE gel (11%), and the gel was divided after electrophoresis. One fraction was stained with CBB (A) and the other was subjected to western blot analysis using anti-*T*. *denticola* antiserum (previously prepared by the authors [8]) (B). The western blot analysis showed two strong positive bands at 53 and 37 kDa, which were predicted to be Msp and TmpC, respectively, by mass spectrometry analysis of the corresponding bands in the CBB-stained gel. The two weak bands (at 91 and 70 kDa) were predicted to be TDE1072 and OppA, respectively. M shows a split band of a molecular-weight standard. The approximate size of each polypeptide is indicated at the right (kDa).

We used two types of SDS-PAGE gels consisting of 11% and 5–20% gradient polyacrylamide. The samples were denatured in a loading buffer consisting of 50 mM Tris/HCl, pH 6.8, containing 1% (w/v) SDS, 0.5 M 2-mercaptoethanol, 10% (w/v) glycerol, and 0.01% bromophenol blue (final concentrations) at 100°C for 5 min, unless otherwise noted. SDS-PAGE gels were stained with Coomassie brilliant blue R-250 (CBB). For western blot analysis, the protein bands in the gel were transferred to a PVDF membrane. The membrane was blocked with 5% skim milk in Tris-buffered saline (TBS), pH 7.5, supplemented with 0.05% Tween 20. We used anti-*T*. *denticola* [8] and anti-TmpC (as described above) antisera as first antibodies. After the membrane was incubated with the first antibodies, it was incubated with a peroxidase-conjugated secondary antibody. Reacted bands were detected using two methods; (i) a chromogenic substrate [0.05% (w/v) 4-chloro-1-naphthol] in TBS supplemented with hydrogen peroxide was used in Fig. 1, and (ii) Amersham ECL Prime Western Blotting detection reagent (GE Healthcare UK Limited, Buckinghamshire, UK) was used in all other experiments.

### Mass spectrometry (MS) analysis

Protein bands detected using SDS-PAGE were identified by matrix-assisted laser desorption/ionization-time-of-flight (MALDI-TOF) MS [24]. After in-gel tryptic digestion, the peptides were extracted, desalted, and analyzed using 4800 MALDI TOF/TOF Analyzer or 4800 Plus MALDI TOF/TOF Analyzer (AB Sciex, Framingham, MA, USA). The identity of the proteins was deduced from the MS peaks by comparative analysis of the mass of each protein with the Mascot database (http://www.matrixscience.com/). Annotation and homology searches were performed using BLAST.

### Construction of gene-deletion mutants

We constructed two mutants of *T*. *denticola* ATCC 35405, Δ*tmpC* and Δ*msp,* in which the entire open reading frames of *tmpC* and *msp*, respectively, had been deleted. Briefly, a *tmpC* (TDE1950)-deletion cassette was constructed using a PCR-based overlap-extension method [25]. The primers used are shown in Table 1. The 526-bp upstream region (ending immediately before the translation initiation codon; primers tmpCU-F/tmpCU-R) and the 533-bp downstream region (beginning immediately following the terminating nonsense codon; primers tmpCD-F/tmpCD-R) of the *tmpC* gene were amplified by PCR from the chromosomal DNA of ATCC 35405, and the 1,036-bp region containing the *ermB* gene (primers ermB-F/ermB-R) was amplified from pVA2198 [26]. These 3 amplicons were fused into a single fragment using the overlap extension method [25] using the primers tmpCU-F and tmpCD-R. The final 2,095-nucleotide fragment was cloned into a pGEM-T Easy vector, and its authenticity was verified by DNA sequencing. Because we could not construct an *msp* (TDE0405)-deletion cassette using a PCR-based method, Eurofins MWG Operon (Huntsville, Alabama, USA) synthesized a gene-deletion cassette that consisted of the 602-bp upstream region (ending immediately before the translation initiation codon) and the 541-bp downstream region (beginning immediately after the terminating nonsense codon) of the *msp* gene, and the 1,036-bp region containing the *ermB* gene, which was cloned in a plasmid vector. These plasmid constructs (50 µg) were linearized by digestion with restriction enzymes, then electroporated into *T*. *denticola* ATCC 35405, as described in our previous paper [8]. Possible transformants were selected in semisolid mGAM-TS containing 0.8% agar supplemented with 40 µg/ml erythromycin. Gene deletions were confirmed by PCR (data not shown).

### Semiquantitative analysis of transcript level

Total RNA was isolated using ISOGEN (Nippon Gene Co., Ltd., Tokyo, Japan) and treated with RNase-free recombinant DNase I (Takara Bio Inc., Otsu, Japan) to eliminate contaminating genomic DNA. The purified RNA (100 ng) was used to generate cDNA with a PrimeScript RT-PCR Kit and Random 6 mers (Takara Bio Inc.). The resulting cDNA was used as a template for the subsequent PCR. The primer sets (i) 1949-F and 1949-R, (ii) 0406-F and 0406-R, and (iii) 1072-F and 1072-R were used to examine the transcription of TDE1949 (immediately downstream gene of *tmpC*), TDE0406 (immediately downstream gene of *msp*), and TDE1072, respectively (Table 1). After standard 25, 27, 29, 31, and 33-cycle PCR, the transcript levels were determined by quantifying the target band intensity after agarose electrophoresis and ethidium bromide staining of the gel.

### Slide agglutination and immunofluorescence assays

The cell-surface exposure of TmpC was examined using slide agglutination and immunofluorescence assays. The slide agglutination assay was performed following a standard protocol. Briefly, *T*. *denticola* cells were washed in PBS, pH 7.4, and the OD_600_ was adjusted to 0.5. The bacterial cells were mixed with anti-TmpC or anti-*T*. *denticola* antiserum on a slide glass, and agglutination was determined. The immunofluorescence assay was performed as described previously [8]. Briefly, *T*. *denticola* culture was placed in the wells of a filtration plate (MultiScreen-GV, 96-well filter plate, 0.22-µm pore size, Millipore Corporation, Billerica, MA, USA). The solutions were removed from the wells through a membrane by centrifugation at 2,000×*g* for 3 min. The wells were blocked with TBS containing 3% (w/v) bovine serum albumin (BSA) at room temperature for 15 min and were incubated with antisera (1,000-fold dilution in TBS containing 1% BSA) for 30 min at room temperature. After washing three times with TBS, the Alexa Fluor 488-conjugated goat IgG fraction to the rabbit IgG secondary antibody (1,000-fold dilution in TBS containing 1% BSA; Thermo Fisher Scientific Inc., Waltham, MA, USA) was added and incubated for 15 min at room temperature in the dark. After washing, the cells were suspended in a small volume of TBS, placed on a slide glass and mounted using ProLong Gold antifade reagent (Thermo Fisher Scientific Inc.). The stained cells were observed by confocal laser scanning microscopy (LSM 710, Carl Zeiss, Oberkochen, Germany).

### Electron microscopy

*T*. *denticola* cells were negatively stained with 1% ammonium molybdate, pH 7.0, and observed using a JEM-1210 transmission electron microscope (JEOL, Tokyo, Japan). We also observed *T*. *denticola* using a scanning electron microscope (JXA-8530FA, JEOL). *T*. *denticola* cells were cultivated on a cover glass, and fixed and dehydrated following a standard method. Cells were coated with 5 nm of platinum and observed.

### Cell surface hydrophobicity

The cell surface hydrophobicity was examined as described previously [27], [28]. Briefly, the washed cells were suspended in an aqueous buffer consisting of 97 mM K_2_HPO_4_, 53 mM KH_2_PO_4_, 30 mM urea, and 0.8 mM MgCl_2_ (pH 7.1), and the cell turbidity was adjusted to OD_400_ 0.5. Aliquots (1.2 ml) were vigorously mixed with *n*-hexadecane (0.6 ml) for 60 s. The OD_400_ of the aqueous phase was measured, and the relative hydrophobicity of the cell surface was calculated using the following formula: % hydrophobicity = [(OD_400_ before mixing OD_400_ after mixing)/OD_400_ before mixing]×100.

### Autoagglutination assay

*T*. *denticola* cells were suspended in PBS, pH 7.4, supplemented with 1 mM CaCl_2_ and 1 mM MgCl_2_, and adjusted to OD_600_ 0.5. Five milliliters of the cell suspension was aliquoted in a 13-mm test tube and was kept static at room temperature. The suspension (0.1 ml) was intermittently collected from the surface, and the OD_600_ value was measured.

### Adherence to gingival epithelial cells

The adherence of *T*. *denticola* to human gingival epithelial cells, Ca9-22 (RIKEN BioResource Center), was examined as described previously [8]. Briefly, *T*. *denticola* cells at OD_600_ 1.0 (corresponding to 5×10^9^ cells/ml) were added to the Ca9-22 monolayer in 8-well Lab-Tek II chamber slides (Thermo Scientific) and incubated at 37°C for 1 h under 5% CO_2_. Then the cells were fixed with 4% paraformaldehyde, pH 7.0, stained with fluorescence-conjugated antibody, and observed by confocal laser scanning microscopy. *T*. *denticola* cells that adhered to the epithelia were counted in the captured images, in which a field corresponded to 0.017 mm^2^.

### Protease activity assay

This assay was performed as described previously [27], [29], with modifications. Briefly, *T*. *denticola* cells were collected when the OD_600_ reached between 0.2 and 0.4, and adjusted to an OD_600_ of 0.2 with fresh mGAM-TS. The aliquots (0.5 ml) were separated into the bacterial pellet and the supernatant by centrifugation at 3,000×*g* for 10 min. The cell pellets were suspended in a double volume (1 ml) of 50 mM Tris/HCl, pH 7.5, supplemented with 2 mM dithiothreitol and 150 mM NaCl. The supernatants (approximately 0.5 ml) were filtrated to remove the remaining cells and mixed with the same volume (approximately 0.5 ml) of 100 mM Tris/HCl, pH 7.5, supplemented with 4 mM dithiothreitol and 150 mM NaCl. Finally, both cell and supernatant samples were prepared with the same dilution rate from the culture. It should be noted that the Tris/HCl, dithiothreitol, and NaCl were at the same concentration in both samples. The synthetic chromogenic substrates for chymotrypsin [N-Succinyl-Ala-Ala-Pro-Phe *p*-nitroanilide (SAAPFNA)] and trypsin [*N*α-Benzoyl-_DL_-arginine 4-nitroanilide hydrochloride (BAPNA)] were obtained from Sigma-Aldrich. The samples (120 µl) were mixed with 30 µl of 5 mM SAAPFNA or 5 mM BAPNA (1 mM at final concentration) and incubated for 1 h at 37°C; the reaction was then stopped by the addition of 50 µl of 20% (v/v) acetic acid. The OD_405_ (via the release of *p*-nitroaniline) was measured. A blank value (without bacterial samples) was subtracted from each value.

### Enzymatic activity assay by API ZYM

A variety of enzymatic activities were semiquantitatively examined by API ZYM (bioMérieux, Inc., Durham, NC, USA) according to the manufacturer’s instruction manual. This kit can detect the following enzymes: alkaline phosphatase, esterase (C4), esterase lipase (C8), lipase (C14), leucine arylamidase, valine arylamidase, cystine arylamidase, trypsin, chymotrypsin, acid phosphatase, phosphohydrolase, α-galactosidase, β-galactosidase, β-glucuronidase, α-glucosidase, β-glucosidase, *N*-acetyl-β-glucosaminidase, α-mannosidase, and α-fucosidase.

### Motility

*T*. *denticola* culture was adjusted to OD_600_ 0.2 with fresh mGAM-TS. The cell suspension (1 µl) was carefully placed on a semisolid mGAM-TS plate containing 0.5% (w/v) agar. The plate was anaerobically incubated at 37°C for one week, and the turbid plaques were measured as an index of bacterial motility.

### Subcutaneous injection of *T*. *denticola* strains into mice

*T*. *denticola* strains were washed in PBS, pH 7.4, and adjusted to OD_600_ 2.0, then subcutaneously injected at 0.1 ml (corresponding to 1×10^9^ bacterial cells) into female 5-week old Balb/c mice. Alternatively, PBS (0.1 ml) without bacteria was injected as a negative control. Each group contained 6 mice. Body weight, appearance, and abscess formation were monitored daily for 3 weeks. Additionally, blood was collected weekly from the peri-orbital sinus and the serum fraction was stored at –20°C until use.

### Enzyme-linked immunosorbent assay (ELISA)

ELISAs were performed as previously described [30]. Briefly, 100 µl of the whole-cell lysate of *T*. *denticola* WT (50 µg/ml protein) was incubated in the wells of an ELISA plate. After the wells were blocked with 3% BSA-containing TBS, the following series of reactions was performed; mice sera (1∶1,000), HRP-conjugated anti-mouse IgM, IgG1, IgG2a, IgG2b, and IgG3 antibodies (1∶5,000) (Bethyl Laboratories, Inc., Montgomery, TX, USA), followed by reaction with *o*-phenylenediamine and H_2_O_2_ in citrate buffer (pH 5.0). The reaction was terminated with 1 M H_2_SO_4_ and the OD_490_ (reference OD_620_) was measured using a microplate reader to determine the antibody titer.

### Macrophage stimulation assay

We examined macrophage activation by measuring TNF-α secretion, as described previously [30]. Briefly, mouse macrophage-like J774-1cells (RIKEN BioResource Center), in RPMI 1640 medium supplemented with 10% (v/v) fetal bovine serum (FBS, LPS-free, heat-inactivated), 100 U/ml penicillin G and 100 µg/ml streptomycin, were seeded at 2.0×10^3^ cells in a well of a 96-well plate and incubated for 24 h at 37°C under 5% CO_2_. *T*. *denticola* cells were harvested and suspended in RPMI 1640 medium with 10% FBS without antibiotics. The bacterial envelope fractions, prepared as described above, were washed and suspended in the medium. We also used LPS (*E*. *coli* O55 LPS, Wako, Osaka, Japan) as a positive control. *T*. *denticola* viable cells, the envelope fractions, or 25 ng/ml LPS was added to J774-1 cells. The supernatants were intermittently collected and filtrated by 0.22-µm membrane to remove macrophage and *T*. *denticola* cells. The TNF-α secreted into the medium was measured using the Mouse TNF alpha ELISA Ready-SET-Go! Kit (eBioscience, San Diego, CA). The envelope fraction extracted from 1 ml of *T*. *denticola* cells (OD_600_ 1.0) contained 1.2 mg of protein. Therefore, we used the envelope fraction at 1.2, 12, and 120 µg/ml protein, corresponding to the cell density at OD_600_ 0.001, 0.01, and 0.1, respectively.

### Statistical analysis

For ANOVA analysis, when *P*<0.05, pairwise comparisons against the control group (WT) were performed. Corrections for multiple comparisons were made according to Dunnett’s test.

## Results

### Identification of major antigenic proteins and construction of mutants

**Figure 2:**
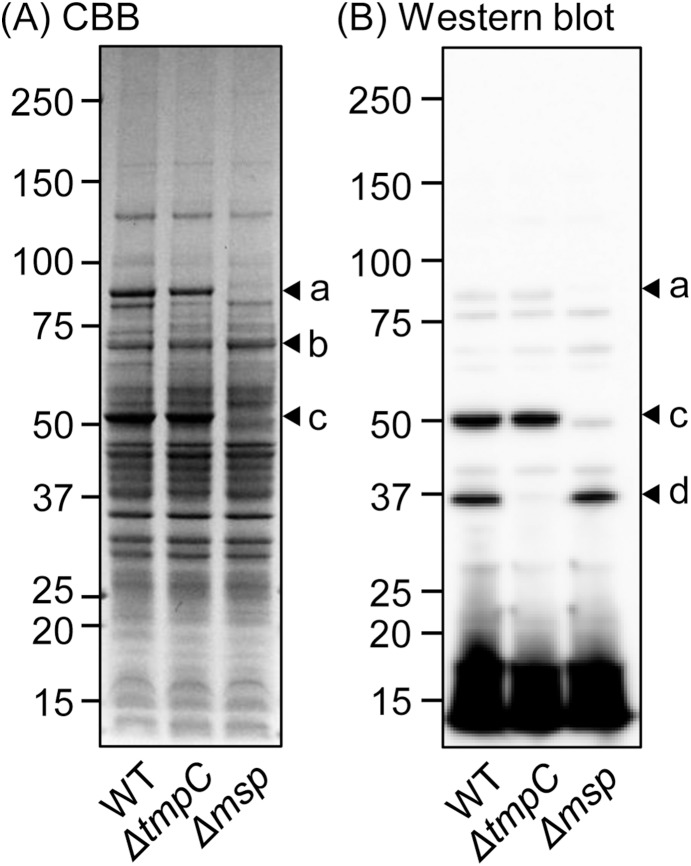
Protein patterns of *T*. *denticola* strains. The whole-cell lysates of the wild type (WT), Δ*tmpC,* and Δ*msp* were applied to SDS-PAGE (5–20% gradient gel) followed by CBB-staining (A) and western blotting using anti-*T*. *denticola* antiserum (B). Panel (A) shows that TDE1072 (a) and Msp (c) were absent in Δ*msp*. Western blot analysis confirmed deficiencies of TmpC (d) and Msp (c) in Δ*tmpC* and Δ*msp*, respectively. The arrowhead b in panel A was identified as OppA. Molecular-weight standards (kDa) are shown at the left.

We applied the envelope fraction instead of whole bacterial cell in order to identify major antigenic proteins much easier by mass spectrometry. The envelope fraction of *T*. *denticola* WT was applied to 2 lanes of an SDS-PAGE gel. After electrophoresis, the lanes were split, and one fraction was used for CBB-staining, and the other was used for western blot analysis using anti-*T*. *denticola* antiserum (Fig. 1). Western blot analysis revealed 4 distinguishable bands. CBB-stained bands corresponding to the bands revealed by western blot analysis were identified by MS. The intense bands at 53 and 37 kDa observed in the western blot analysis were predicted to be Msp and TmpC, respectively. The weak bands at 91 and 70 kDa were predicted to be TDE1072 protein and OppA, respectively. We showed that the two intensely immunoreactive bands were Msp and TmpC, using gene-deletion mutants (Fig. 2); the 53- and 37-kDa bands were absent in Δ*msp* and Δ*tmpC*, respectively. Additionally, TDE1072 protein band was not observed in Δ*msp* (Fig. 2A). Signals below 15 kDa in western blot analyses were observed. The area around 15kDa likely includes lipids, small size proteins, materials degraded during preparation.

We examined a potential polar effect in the mutants. The TDE1949 gene (a downstream gene of *tmpC*) in Δ*tmpC* and the TDE0406 gene (a downstream gene of *msp*) in Δ*msp* were transcribed at quantities comparable to those of WT (data not shown), suggesting that there is little polar effect on transcription. In addition, the transcript level of the TDE1072 gene was similar in WT and Δ*msp*, although the protein band was missing in the mutant, as described above (data not shown).

### Cellular localization and conformation of Msp and TmpC

**Figure 3:**
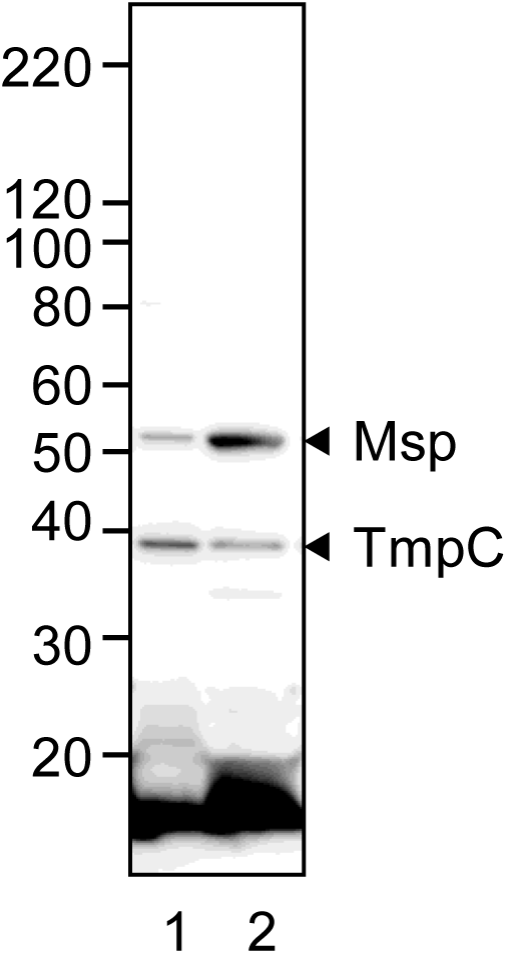
Cellular localization of TmpC and Msp proteins. *T*. *denticola* cells were fractionated into the insoluble (lane 1) and soluble fractions (lane 2) by suspending in 0.1% Triton X-100. The samples were applied to SDS-PAGE (5–20% gradient gel) followed by western blot analysis using anti-*T*. *denticola* antiserum. A molecular-weight standard (kDa) is shown at the left.

We have previously reported that both Msp and TmpC are localized to the envelope fraction, including the inner and outer membrane fractions (Fig. 4C in [8]). The present study further examined their localization by fractionating the cell surface layer, which primarily contains the outer membrane. Fig. 3 shows that Msp was primarily detected in the outer membrane fraction. However, although substantial TmpC was detected in the outer membrane, more TmpC was detected in the cell fraction, suggesting that TmpC localized in the inner membrane. Additionally, it appears that TmpC is not exposed on the cell surface, because anti-TmpC antiserum did not cause agglutination with intact *T*. *denticola* cells and did not produce a clear signal in the immunofluorescence assay, although anti-*T*. *denticola* antiserum did both (data not shown).

**Figure 4:**
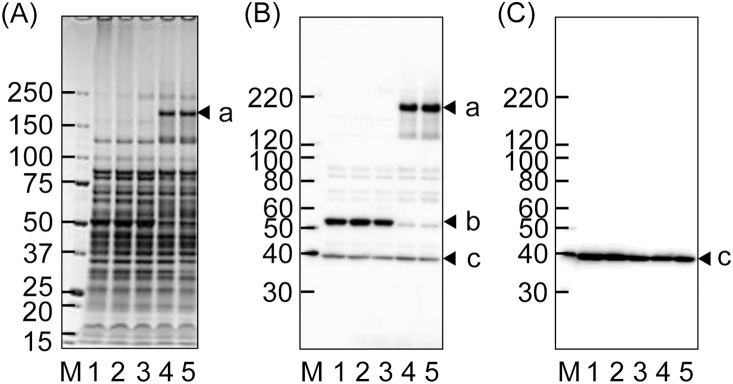
Effect of temperatures on mobility of TmpC and Msp in SDS-PAGE. The whole-cell lysate of the wild type of *T*. *denticola* was mixed with a loading buffer for SDS-PAGE, and heated at 100°C (lane 1), 80°C (lane 2), 60°C (lane 3), or 37°C (lane 4) for 10 min. Samples in lane 5 were not heated (maintained at room temperature). The SDS-PAGE gels (5–20% gradient) were subjected to CBB-staining (A), and western blotting using anti-*T*. *denticola* antiserum (B) and anti-TmpC antiserum (C). Arrowheads indicate conformational Msp (a), monomeric Msp (b), and monomeric TmpC (c). Msp, but not TmpC, appears to form a complex. A molecular-weight standard (lane M, kDa) is shown at the left of each panel.

We examined thermal denaturation of proteins in SDS-PAGE (Fig. 4). It has been reported that Msp forms a complex with other molecules such as dentilisin [31], [32], but no studies have reported on the conformation of TmpC. A complex form of Msp was detected at approximately 200 kDa (arrowhead a in Fig. 4) in denaturing conditions at less than 37°C, but it was almost completely dissociated into the monomer (53 kDa) by heating at above 60°C, which is consistent with a previous report [33]. MS analysis detected two *T*. *denticola* proteins, Msp and cytoplasmic filament protein A (CfpA, TDE0842), in the band indicated by arrowhead a in Fig. 4A. TmpC only produced a band at the monomer position (38 kDa), with the same intensity regardless of temperature (Figs. 4B and C), indicating that TmpC does not form a complex in these conditions.

### Physiological and pathological characteristics of the mutants in *in* *vitro* experiments

No significant differences in growth were observed among strains (data not shown). Additionally, transmission and scanning electron microscopy showed no morphological changes in the mutants (data not shown).

**Figure 5:**
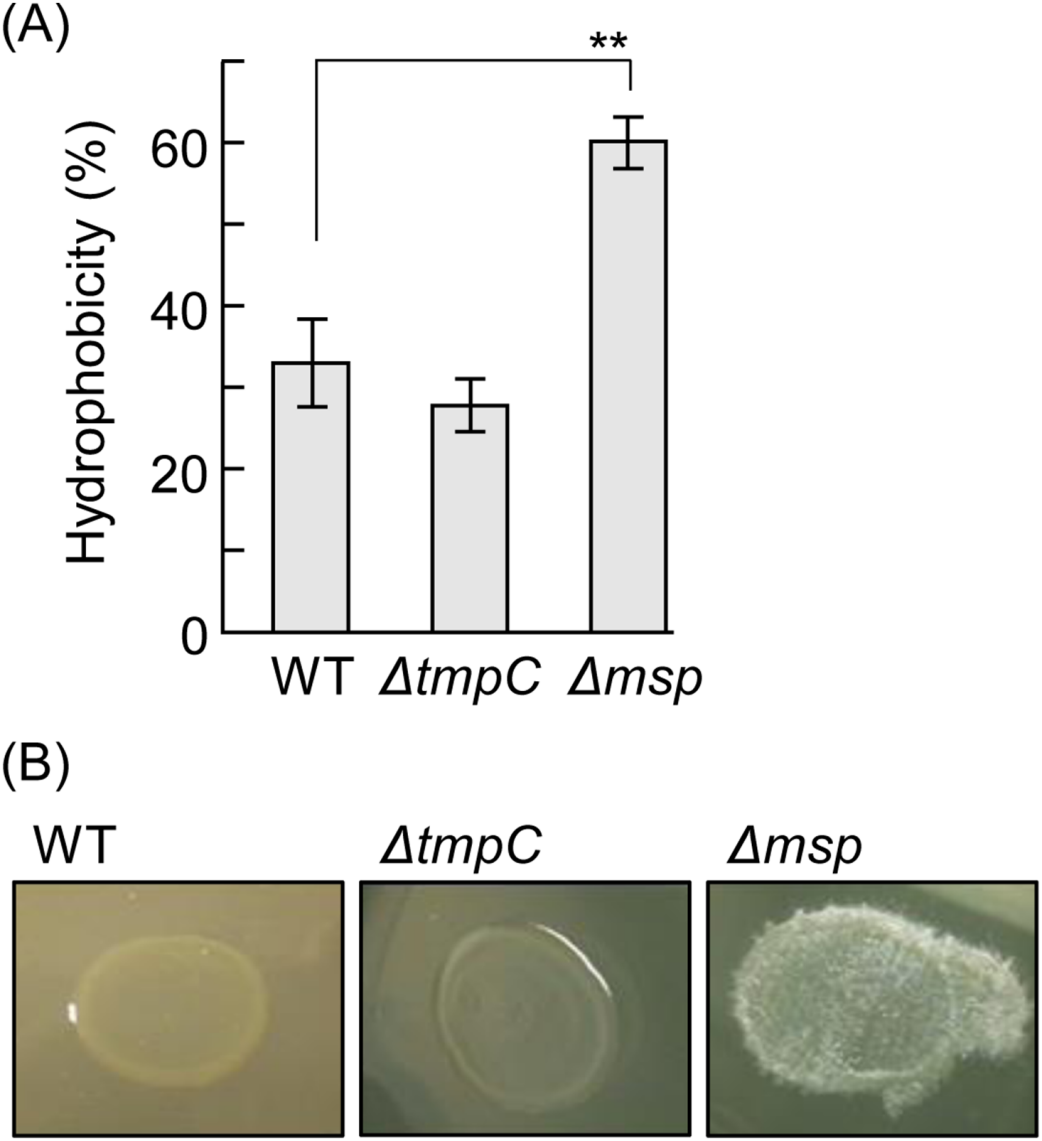
Cell surface hydrophobicity. (A) Cell surface hydrophobicity was quantified by measuring the partition coefficient of *T*. *denticola* cells between the aqueous and *n*-hexadecane phases. The data presented show a representative result from two independent experiments and are expressed as the means ± SD (n = 3). A Dunnett’s test was performed to compare the wild type and mutants (**, *P*<0.01). (B) Colony appearance of *T*. *denticola* strains cultivated on a medium containing 2.5% agar. The wild type and Δ*tmpC* were smooth in appearance, whereas Δ*msp* were rough and clearly different, indicating that Δ*msp* has a hydrophobic surface.

**Figure 6:**
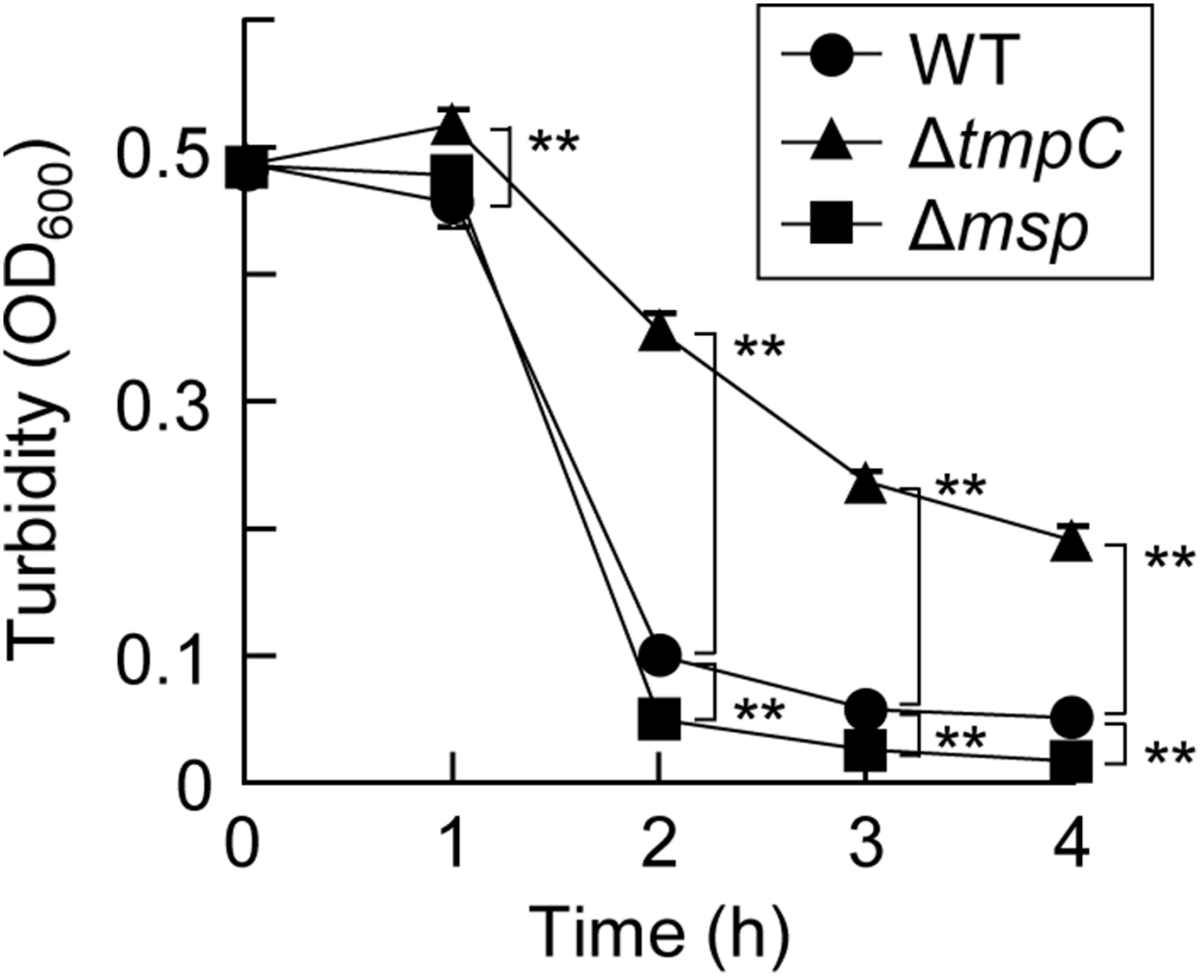
Autoagglutination assay. Autoagglutination was examined by monitoring the OD_600_ of the surface of the cell suspension. The data presented show a representative result from three independent experiments and are expressed as the means ± SD (n = 3). A Dunnett’s test was performed to compare the wild type and mutants at each time point (**, *P*<0.01).

Because both Msp and TmpC were detected in the envelope fraction, we examined the cell surface properties of the mutants. Δ*msp* markedly increased cell surface hydrophobicity (Fig. 5A). Fig. 5B shows that WT and Δ*tmpC* expressed smooth colony forms, whereas the colony form of Δ*msp* was rough, suggesting that the surface of Δ*msp* became more hydrophobic. We monitored the precipitation of the bacterial suspension to examine its autoagglutination ability (Fig. 6). Autoagglutination of Δ*tmpC* significantly slowed, whereas the Msp defect augmented autoagglutination, although it did not differ much from WT. We next examined the adherence of *T*. *denticola* to gingival epithelial cells (Fig. S1). All strains exhibited substantial adherence, but no significant differences were observed between the strains.

**Figure 7:**
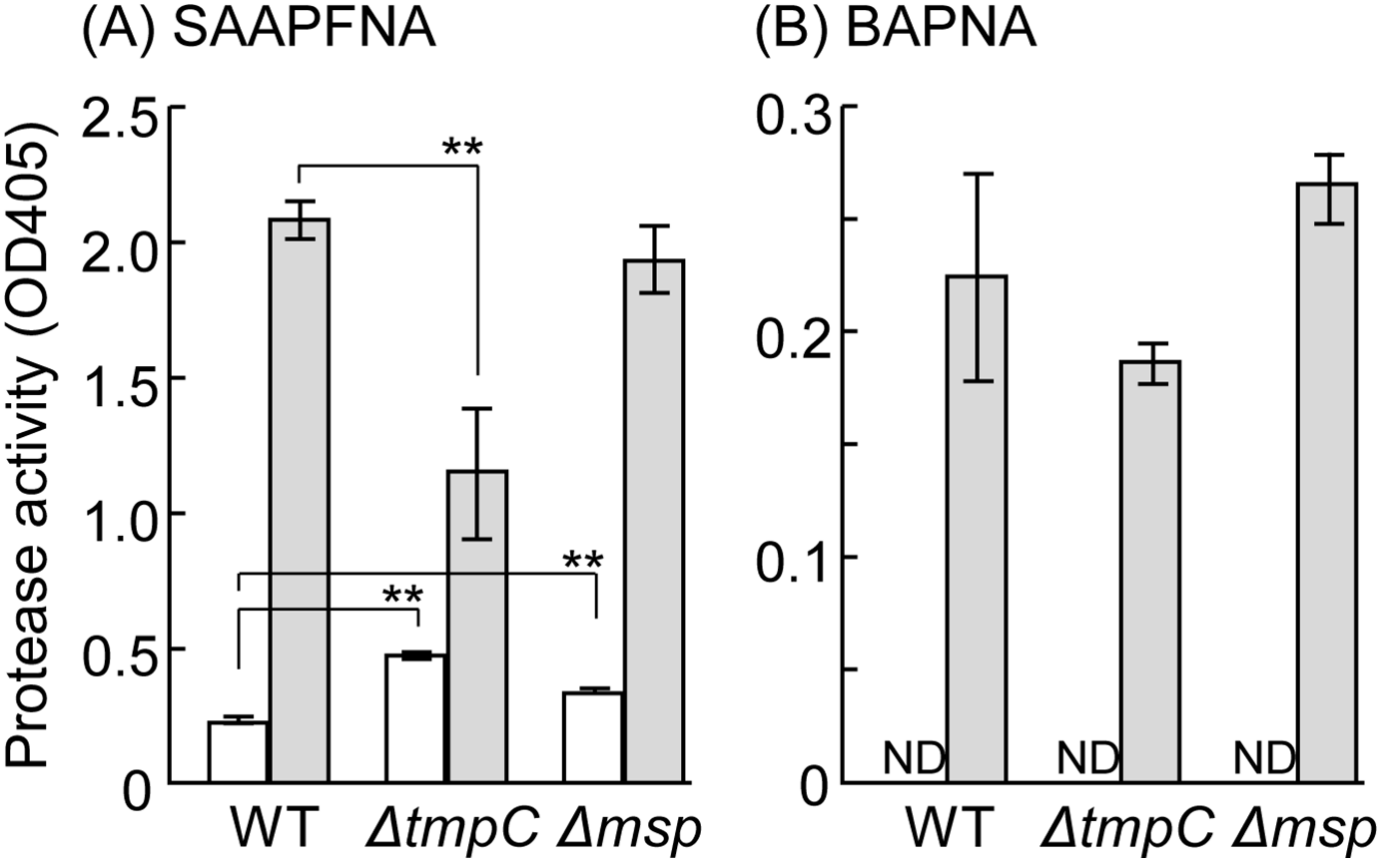
Protease activity assay. *T*. *denticola* cultures were separated into supernatant (white) and cell (grey) fractions, and diluted to an equivalent concentration. SAAPFNA (A) and BAPNA (B) were used as a substrate; these are typical substrates for chymotrypsin and trypsin, respectively. ND indicates that the result was below the detectable level. The data presented show a representative result from two independent experiments and are expressed as the means ± SD (n = 3). A Dunnett’s test was performed to compare the wild type and mutants (**, *P*<0.01). Note that protease activities (OD_405_ values) assayed using BAPNA were approximately 10-fold lower than those assayed using SAAPFNA.

We measured the protease activity in the cell-associated and the culture supernatant fractions (Fig. 7). In the cell-associated fraction, Δ*tmpC* showed significantly lower protease activity for SAAPFNA, compared to WT. In contrast, the supernatant of Δ*tmpC* showed significantly higher activity than WT, although the difference was only modest. Similarly, the supernatant of Δ*msp* showed significantly higher activity than WT, but the difference was small. Protease activity to the trypsin substrate BAPNA was detected only in the cell-associated fraction, and the activity was much lower than the SAAPFNA hydrolysis activity; and significant differences were not detected between strains, although a pattern similar to that of SAAPFNA activity was observed.

In the API ZYM assay, a positive reaction was detected in WT for alkaline phosphatase, esterase (C4), esterase lipase (C8), leucine arylamidase, trypsin, chymotrypsin, acid phosphatase, phosphohydrolase, and α-galactosidase. Δ*tmpC* and Δ*msp* exhibited profiles similar to that of the WT, except that Δ*tmpC* showed a stronger color reaction in α-galactosidase test; WT and Δ*msp* were score 3 while Δ*tmpC* was score 5.

**Figure 8:**
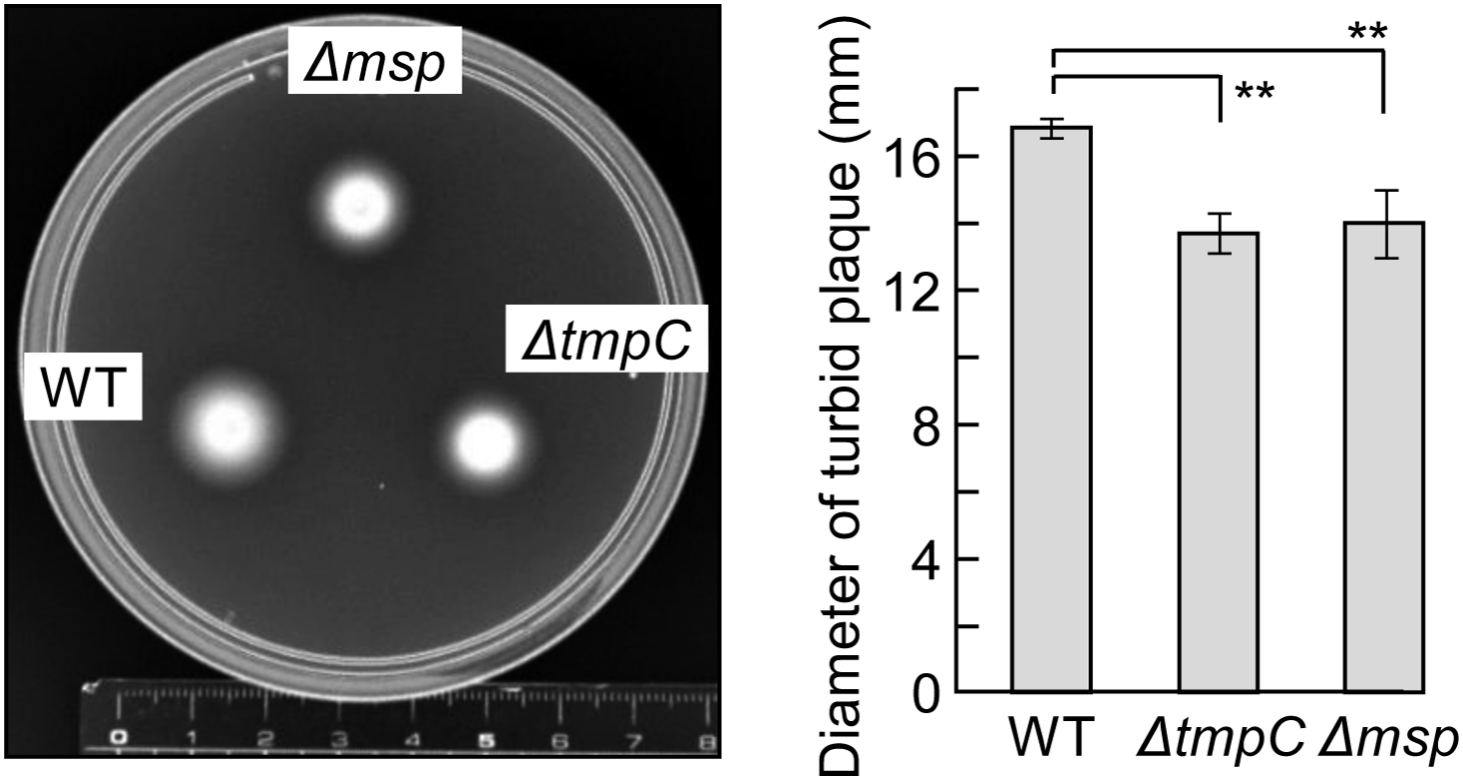
Motility assay. *T*. *denticola* strains were seeded on a medium containing 0.5% agar. After the plate was anaerobically incubated at 37°C for one week, the turbid plaque was measured. Each plate presented is a representative image. The data are expressed as the means ± SD (n = 3). A Dunnett’s test was performed to compare the wild type and mutants (**, *P*<0.01).

We examined bacterial motility in a semisolid medium (Fig. 8). The diffusion diameter of each mutant was significantly smaller than that of the WT, but the difference was modest.

WT and mutant strains showed identical MICs, as follows; 0.025 µg/ml ampicillin, 0.78 µg/ml gentamicin, 25 µg/ml kanamycin, 3.13 µg/ml metronidazole, 0.003 µg/ml penicillin G, 0.39 µg/ml tetracycline, and 3.13 µg/ml vancomycin.

### Challenge with *T*. *denticola* to mice and macrophage-like cells

**Figure 9:**
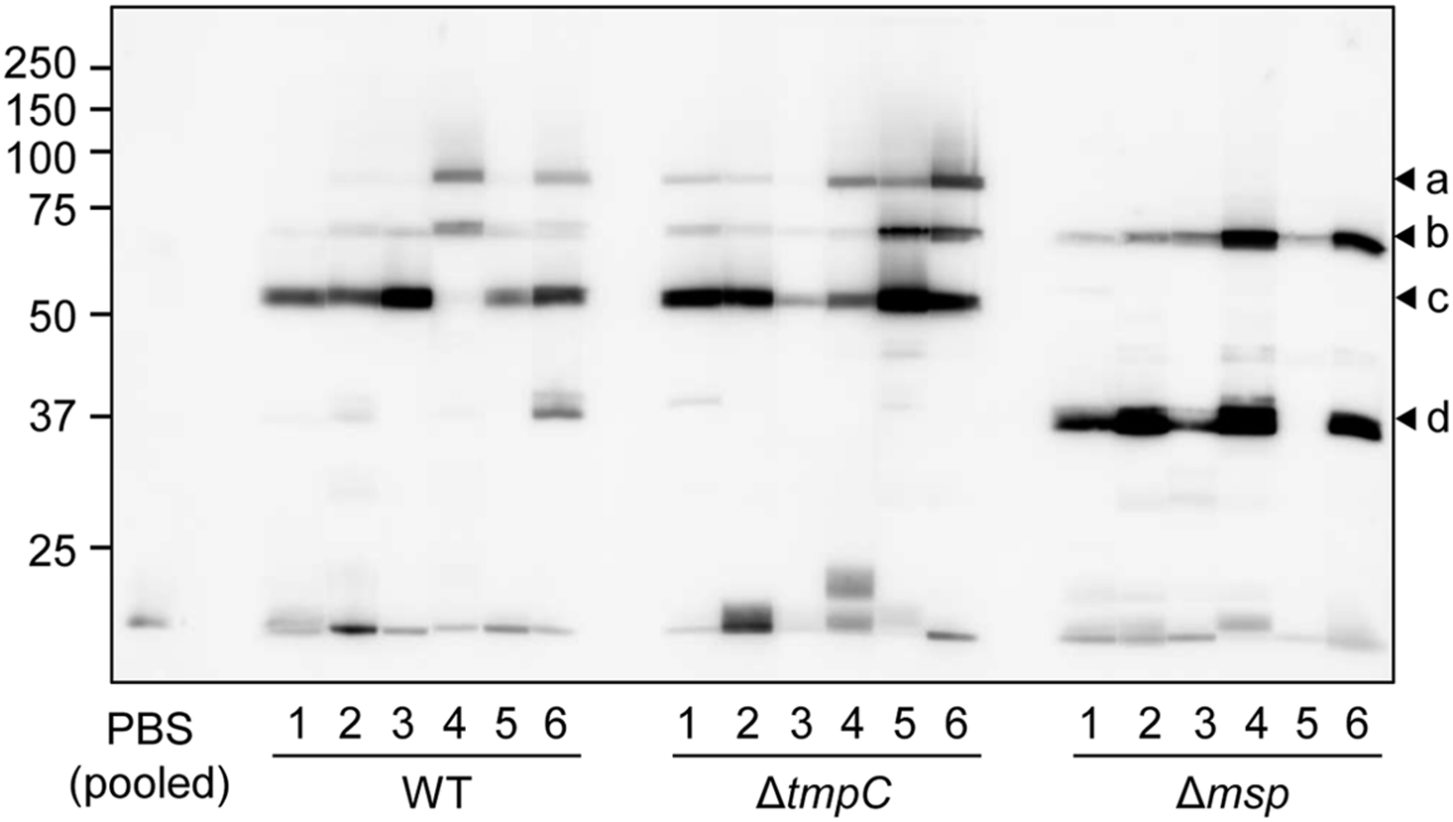
Reactivity of sera of mice injected with *T*. *denticola* strains to cellular components of *T*. *denticola*. The whole-cell lysate of wild-type *T*. *denticola* was applied to SDS-PAGE (11% gel) and blotted on a PVDF membrane. The PVDF membrane was cut into strips and reacted with each serum sample collected at 3 weeks after injection. As a negative control, sera of mice injected with PBS were pooled and subjected to immunoreaction. Numbers denote individual mice. Major bands were indicated by a, b, c, and d and were predicted to react to TDE1072, OppA, Msp, and TmpC, respectively. A molecular-weight standard (kDa) is shown at the left.

Although *T*. *denticola* strains were subcutaneously injected into mice to examine abscess formation, no lesions were observed in any mice during the 3 weeks following the injection. Additionally, no mice showed weight loss or abnormal appearance, such as hair disorder. Sera were collected weekly after the injection and were subjected to immunological analysis. The sera of mice injected with WT reacted strongly to Msp in 5 of 6 mice, whereas a clear band for TmpC was observed in only 1 mouse (mouse #6 of the group injected with WT, #6/WT), a weak band was observed in 3 mice (#1, 2 and 4/WT); no signal was detected in the remaining mice (#3 and 5/WT) (Fig. 9). The bands indicated by arrowheads c and d were confirmed to be Msp and TmpC, respectively, using their gene-deleted mutants as antigens (data not shown). WT injection elicited additional bands at positions corresponding to TDE1072 (4 of 6 mice) and OppA (all mice) although some of the bands were weak intensity. Δ*tmpC* injection induced antibody to Msp in all mice. Bands corresponding to TDE1072 and OppA were also detected. In the case of injection with Δ*msp*, 5 of 6 mice showed a strong reactivity to TmpC, and all samples showed a band corresponding to OppA, whereas no band was observed at the position corresponding to TDE1072. This supports the hypothesis that the band indicated by arrowhead a is TDE1072, because Δ*msp* did not express TDE1072 (see Fig. 2).

Antibody production was quantified in the sera by ELISA and antibody isotypes and subclasses were defined (Fig. S2). A high titer of the IgM antibody was observed in all groups at 1 week after the injection, which subsequently decreased. IgG antibodies of all subclasses tested (IgG1, IgG2a, IgG2b, and IgG3) rose substantially from 2 weeks and increased further at 3 weeks. However, no significant differences were observed between WT and mutants in any antibody class during any periods.

**Figure 10:**
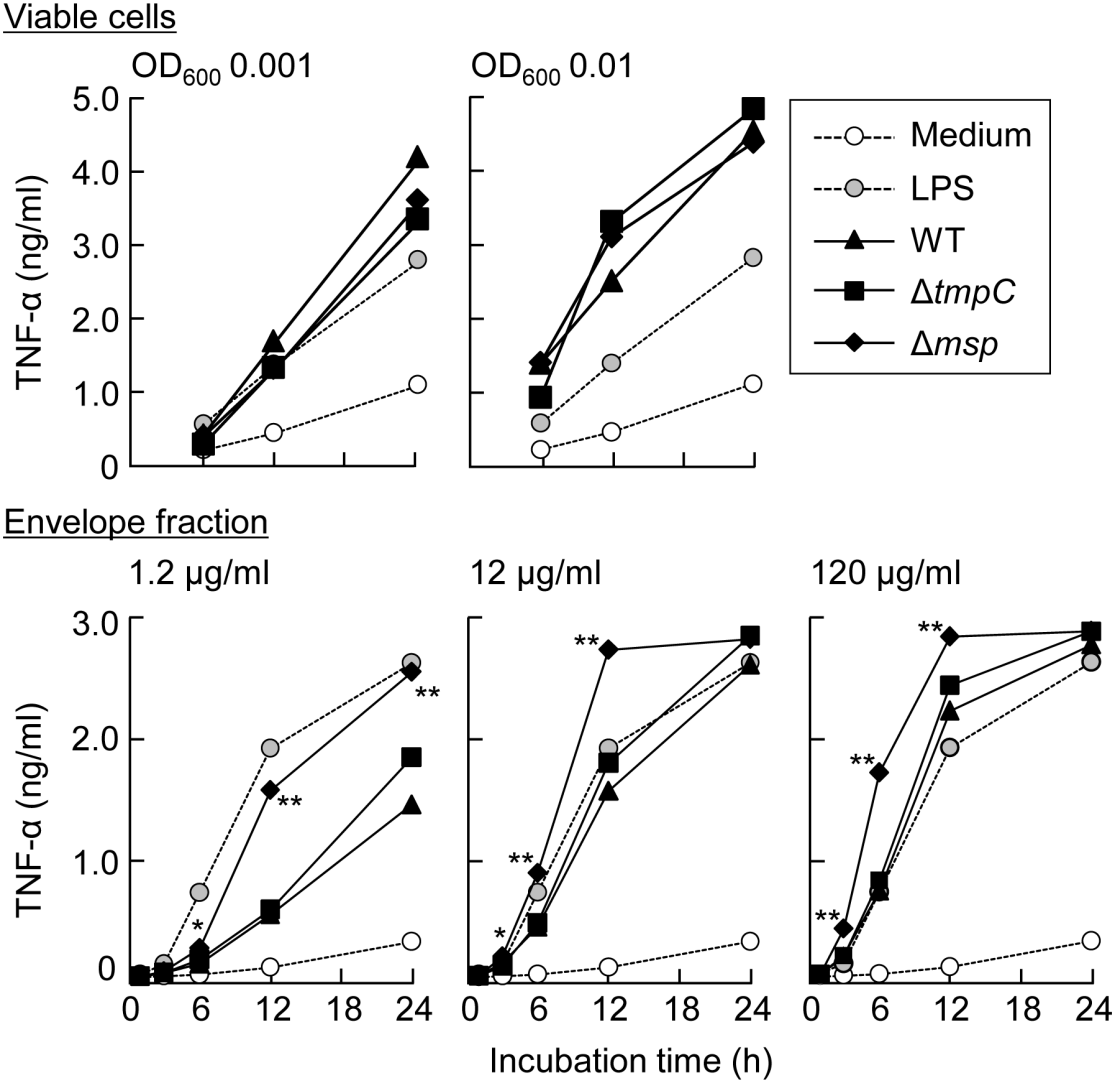
Macrophage stimulation assay. Mouse macrophage-like J774-1cells were incubated with LPS (25 ng/ml, extracted from *E*. *coli*), viable cells of *T*. *denticola* strains (OD_600_ 0.001 and 0.01) and envelope fractions (1.2, 12, and 120 µg protein/ml). TNF-α secreted from the J774-1 cells was measured by ELISA. Medium (open circle) and LPS (grey circle) were used as negative and positive controls, respectively. The data presented show a representative result from two independent experiments and are expressed as the means ± SD (n = 3). A Dunnett’s test was performed to compare the wild type and mutants at each time point (* and **, *P*<0.05 and *P*<0.01, respectively).

We examined immunological stimulation of *T*. *denticola* to macrophage-like cells (Fig. 10). In experiments using viable cells at OD_600_ 0.001 (equal to 5×10^6^ cells/ml), all strains equivalently induced TNF-α secretion from the macrophage-like cells, comparable to administration of 25 ng/ml *E*. *coli* LPS, and cytokine levels increased continuously until 24 h. When the bacterial concentration was increased to OD_600_ 0.01, cytokine secretion was enhanced. Further ten-times increase of the bacterial concentration (OD_600_ 0.1) severely injured the macrophage-like cells (meaning that a number of cells were detached from the plate), and TNF-α production decreased (data not shown). We next used the envelope fractions, because TmpC and Msp were localized in this fraction. The envelope fraction of Δ*msp*, at all concentrations, showed significantly higher stimulatory activity than that of WT, although levels became comparable at 24 h after the addition of 12 and 120 µg/ml protein, probably because TNF-α production reached a plateau. The envelope fraction of Δ*tmpC* produced values equivalent to those of WT in all situations.

## Discussion

Many reports have indicated that Msp is a major antigen in *T*. *denticola*. Umemoto *et al*. [9], [34] and Cockayne *et al*. [12] showed that rabbit sera immunized with *T*. *denticola* cells recognized a protein of approximately 53-kDa as a major antigen, which may be Msp. Veith *et al*. [11] showed that Msp produced the strongest signal in western blot analysis using two-dimensional electrophoresis. Furthermore, Capone *et al*. [10] reported that sera from patients with periodontal disease strongly reacted to Msp. It is reasonable that Msp is a major antigen because it is the most abundant protein in *T*. *denticola*, based on SDS-PAGE patterns, and because Msp, exposed on the cell surface, is easily accessible to immune cells. TmpC, as described in the introduction, has also been reported to be an antigenic protein in *T*. *denticola* [11], and its antigenicity has also been demonstrated in *T*. *pallidum* [20], [21]. We also showed here that rabbit serum immunized with *T*. *denticola* reacted to Msp and TmpC, and that the intensities of the immunoreactive bands were almost equal (Figs. 1 and 2). Our experiments in mice also showed the strong antigenicity of Msp, although an antibody to TmpC was not always induced. However, the reactivity to TmpC was notably increased in sera of Δ*msp*-injected mice, indicating that TmpC has a potential antigenicity, even to mice, although it is unclear why Msp defects enhanced the induction of antibodies to TmpC. It should be noted that TmpC appeared to be expressed in much smaller quantities than Msp in *T*. *denticola* (Figs. 1 and 2), suggesting that TmpC itself may have strong immunogenicity. This may be partially explained by the fact that TmpC was lipidated [20], and lipidation generally enhances immune potency [35]. Antibodies to the TDE1072 protein were also detected in a rabbit and in mice injected with the wild-type *T*. *denticola*. We showed that TDE1072 was a membrane-associated protein [8], annotated as an ABC transporter substrate-binding protein and predicted to be lipidated. The 70-kDa proteins shown in Figs. 1 and 9 were predicted to be OppA, exhibiting antigenicity in a rabbit and in all mice. It has been reported that the 70-kDa protein is likely to be OppA displaying antigenicity [11]. Similar to TmpC, antibody (presumably to OppA) was increased in Δ*msp*-injected mice. Collectively, the TDE1072 protein and OppA appear to be potential antigens in *T*. *denticola*.

We constructed mutants defective in Msp and TmpC in order to examine their functions in *T*. *denticola*. We were not able to confirm the effect of genes via complementation, because we have not established a genetic complementation system in this bacterium although a paper reported it [36]. However, we confirmed the absence of a polar effect on genes immediately downstream of the deleted target genes. Therefore, phenotypic changes in the gene-deletion mutants were likely attributed to gene deletion. However, TDE1072 was undetected in addition to the absence of Msp in Δ*msp*, although transcription of TDE1072 was found to be normal; therefore, the observed phenotypic changes resulted, at least in part, from the loss of the TDE1072 protein.

Δ*msp* and Δ*tmpC* did not show a growth defect or apparent morphological changes, indicating that Msp and TmpC are not essential for bacterial viability, at least in a rich medium. Additionally, the motility of mutants was not strongly affected, although statistical differences were detected. However, Δ*msp* showed a significant change in cell surface hydrophobicity. It is plausible that loss of this abundant protein from the cell surface drastically altered its surface property. Additionally, Δ*msp* was missing another major membrane protein, TDE1072. Autoagglutination of Δ*msp* was slightly increased, which might also have resulted from the increased surface hydrophobicity. In contrast, in Δ*msp*, bacterial adherence to epithelial cells was unaltered, although Msp has been reported to interact directly with host tissues [37]. It is possible that other factors compensated for the adherence of Δ*msp*. It has been reported that OppA functions as an adherent factor [38], although its expression in Δ*msp* was comparable to that of WT (Fig. 2, arrowhead b). On the other hand, TmpC defects did not alter surface hydrophobicity or adherence to epithelial cells. However, Δ*tmpC* exhibited significantly decreased autoagglutination, suggesting that the chemical properties of the cell surface of the mutants were altered, although the changes remain largely unexplained.

Msp has been reported to be a porin [18], [39]. The outer membrane generally functions as a barrier against harmful materials such as antibiotics [40]. However, porin is a major influx pathway for antibiotics, and its deficiency occasionally causes an increase of the MIC [19]. However, Msp defects did not alter the MICs of the antibiotics tested in this study, suggesting that if Msp is a porin, it is not a primary pathway for antibiotic influx in *T*. *denticola*. However, antibiotics may be able to bypass porin and access their target, because the outer membrane of spirochetes seems fragile.

Msp is claimed to form a stable trimer in the outer membrane as well as in the periplasmic space and to be exposed on the cell surface [13], [41], which is basically consistent with the results of this study (Fig. 4). However, our MS analysis detected CfpA as well as Msp in the complex band (arrowhead a in Fig. 4A). Because it has been reported that CfpA forms major cytoplasmic filaments and localizes in the cytoplasm [42], it is unlikely that Msp physically interacts with CfpA. We therefore hypothesize that an oligomeric conformer of CfpA (80 kDa of monomer) concomitantly migrated to the same position as Msp in the SDS-PAGE analysis. However, Msp is predicted to form a complex with dentilisin [31], [41], but no other proteins, including dentilisin, were detected in this study, except CfpA. Ishihara *et al*. [27] reported that Msp did not form a complex with dentilisin. Collectively, these data suggest that Msp forms a homo-oligomer, although further analysis is needed. However, TmpC was likely monomeric. Additionally, TmpC was largely detected in the cell-associated fraction, suggesting that TmpC localized in the inner membrane because TmpC was fractionated in the envelope [8]. Although TmpC was also detected in the outer membrane fraction, its surface exposure was not convincingly detected by the slide agglutination or immunofluorescence assays using anti-TmpC antiserum. It has been reported that in *T*. *pallidum* TmpC is a monomeric lipoprotein associated with the cytoplasmic membrane and not exposed on the cell surface [20]. However, it is necessary to examine by other methods such as immunoelectron microscopy to demonstrate the exact localization.

We examined the activity of chymotrypsin- and trypsin-like proteases using SAAPFNA and BAPNA, respectively, as chromogenic substrates (Fig. 8). Similar to other reports, *T*. *denticola* expressed a strong CTLP activity as compared to trypsin-like protease activity [3]. In addition, protease activity (to both substrates) was largely detected in the cell-associated fraction. Defects of PrtP, a component of the dentilisin complex, abolished the CTLP activity, but did not affect expression of the Msp protein (monomer) [27], [43]. However, CTLP activity in Msp-defective mutants varies in different reports; the mutant, when compared to the WT, either decreased [44], did not change [45], or increased [31]. In this study, we did not detect a significant difference in CTLP activity between WT and Δ*msp*. Our results show that Msp is not necessary to express CTLP activity, although it remains controversial whether Msp forms a complex with dentilisin, as described above. Surprisingly, TmpC defects caused a significant decrease in CTLP activity in the cell fraction. More CTLP may have been released into the medium, because the supernatant of the mutant showed a significantly higher value. However, given the values shown in Fig. 8, the increment of the supernatant was modest and could not compensate for the decline in the cell fraction. Alternatively, TmpC defects might decrease the protein expression of dentilisin, and it is necessary to quantify the expression. API ZYM detected an obvious increase in α-galactosidase activity in Δ*tmpC*. Because *T*. *denticola* possesses only one gene (TDE1453) annotated as α-galactosidase, this result indicates that TmpC defects increased either the protein expression or the activity of the enzyme. However, similar to CTLP, the relationship between TmpC and α-galactosidase remains unclear.

A mouse abscess model is commonly used to evaluate the pathogenicity of periodontal pathogens, including *T*. *denticola* [27], [46], [47]. However, we did not observe any pathological changes, including body weight loss, hair disorders, or abscess formation in any of the mice injected with WT, Δ*msp,* or Δ*tmpC* during the study period. Although this difference is probably attributable to experimental conditions, *T*. *denticola* likely expresses low virulence in this model because other periodontal pathogens, such as *Porphyromonas gingivalis* [48] and *Tannerella forsythia* [49], were shown to cause an abscess under similar conditions in our previous study. However, *T*. *denticola* injection elicited antibodies to the bacterial components, as described above. We then examined a time course and classified the antibodies by ELISA. No significant differences were observed between strains for any class of immunoglobulins during the study period (Fig. S2). All IgG antibody subclasses tested were substantially induced, suggesting that both Th1 and Th2 reactions were evoked in the mice [50]. It should be noted that we did not compare titers between subclasses because antibody concentrations were not expressed as absolute concentrations. Further study in an oral infection model or human samples is required to elucidate pathogenicity and immune responses.

Previous reports have shown opposing results, that *T*. *denticola* immunologically stimulated [51]–[54] or did not stimulate mammalian cells [55]–[57]. We showed here that *T*. *denticola*, both viable cells and the envelope fraction, clearly stimulated TNF-α secretion in macrophage-like cells (Fig. 10). We conclude that *T*. *denticola* has the potential to stimulate mammalian cells. Stimulation by viable *T*. *denticola* cells did not differ between strains. However, a difference was observed for the envelope fraction; Δ*msp* showed significantly higher values than WT at all concentrations. Although it is difficult to explain the difference observed between viable cells and the envelope fraction, we think that the envelope fraction more clearly reflects the influence of the protein defects; for example, the bacterial proteases in the envelope fractions were inactivated by protease inhibitors. It should be noted that the concentration of membrane lipids in the envelope fractions may have differed between strains, because the envelope fraction was prepared by adjusting for protein concentration. Indeed, Msp and TDE1072 proteins comprised 8% and 4%, respectively, (total 12%) of the total amount of whole envelope proteins in WT. Therefore, the envelope fraction from Δ*msp* contained more lipid components (possibly 12% more) than that from WT. Increasing the lipid component might enhance stimulation because the lipid membrane fraction (without proteins) of *T*. *denticola* has been shown to induce cytokine production in macrophage cells [51], [52]. However, Δ*msp* showed a time course similar to a 10-fold higher concentration of proteins from the WT; the response curves for 1.2 and 12 µg/ml protein of Δ*msp* were equivalent to those for 12 and 120 µg/ml protein of WT, respectively. Because the lipid content of Δ*msp* is unlikely to be 10-fold greater than that of WT, we hypothesize that the difference in stimulation between the strains resulted largely from the defects in Msp and TDE1072. However, it has been reported that pure Msp stimulates cytokine secretion [52], [53], which contradicts our result. However, isolated Msp may be more potent than membrane-embedded Msp. As is so often the case between a host and parasitic bacteria, Msp may mask the immunogenicity of viable *T. denticola* cells. Further experiments are necessary to elucidate the role of Msp in the immune response.

In conclusion, the major antigenic proteins Msp and TmpC affect the physiological and pathological properties of *T*. *denticola*, especially cell surface properties. Further study is needed to elucidate their functions more clearly. In addition, their effects on immune responses, related to their strong immunogenicity, may imply a significant finding to understand the mode of bacterial infection.

## Supporting Information

Figure S1 **Adherence assay of** ***T*** **.** ***denticola*** **strains to gingival epithelial cells.** *T*. *denticola* cells were incubated with Ca9-22 cells for 1 h, then visualized by immunostaining. The left panel shows a representative image of wild-type *T*. *denticola* (green) adhered to Ca9-22 cells (red). *T*. *denticola* cells were counted to quantify their adherent activity. The data presented show a representative result from four independent experiments and are expressed as the means ± SD (n = 30). No significant differences between strains were observed (ANOVA).
(TIF)

Figure S2 **Antibody titer of sera of mice injected with** ***T*** **.** ***denticola*** **strains.** Antibody (IgM, IgG1, IgG2a, IgG2b, and IgG3 antibodies) titer of sera of mice injected with *T*. *denticola* strains to the whole-cell lysate of the wild type was measured by ELISA. The data are expressed as the means ± SD (n = 6 mice). No significant differences between strains were observed at any point (ANOVA).
(TIF)
