# Supplementary figures and images for: Characterization of Treponema denticola Mutants Defective in the Major Antigenic Proteins, Msp and TmpC
Source: PLoS One. 2014 Nov 17;9(11):e113565. doi: 10.1371/journal.pone.0113565 (PMC4234677; doi:10.1371/journal.pone.0113565)

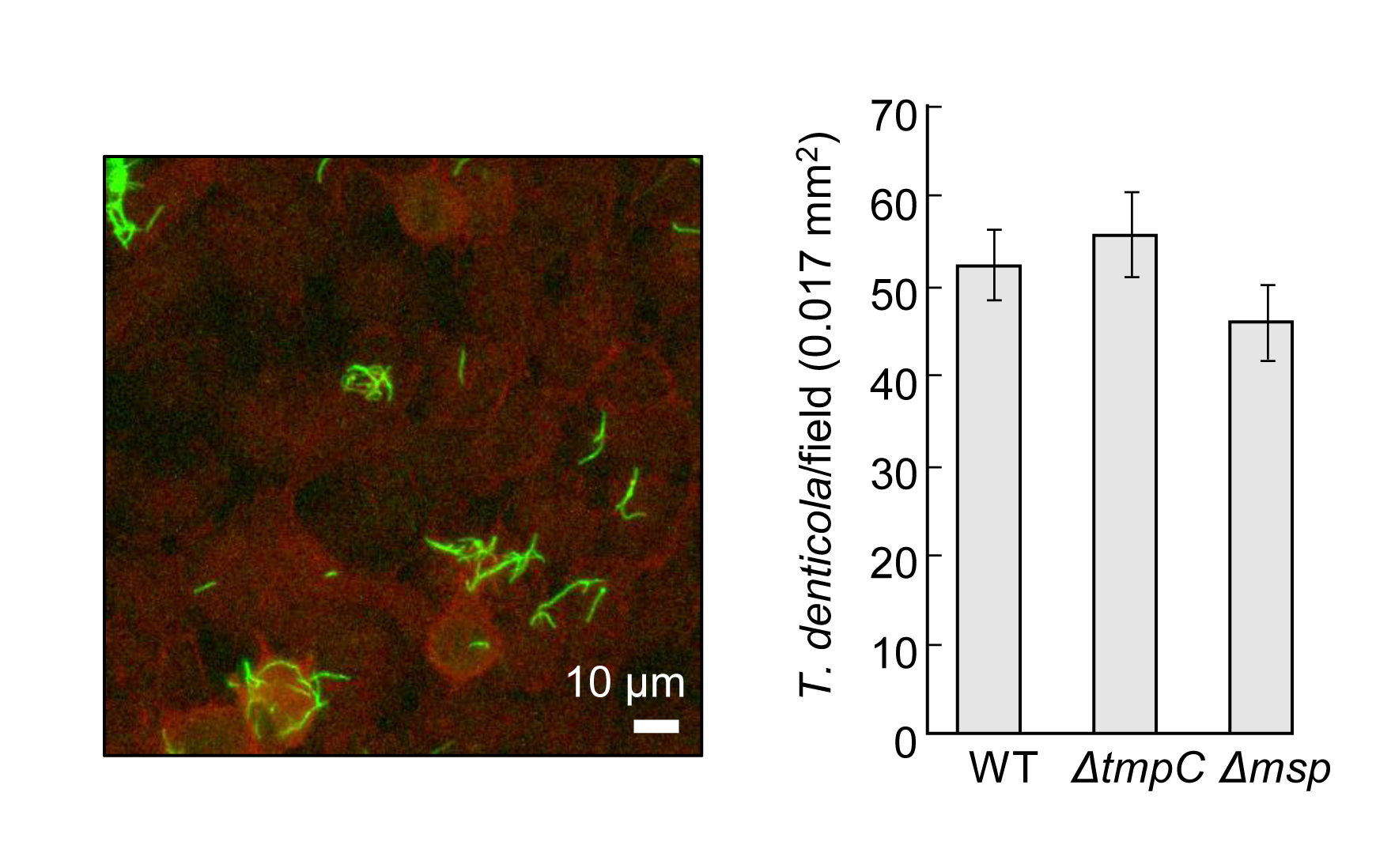

Supplement: Figure S1 — Adherence assay of T . denticola strains to gingival epithelial cells. T. denticola cells were incubated with Ca9-22 cells for 1 h, then visualized by immunostaining. The left panel shows a representative image of wild-type T. denticola (green) adhered to Ca9-22 cells (red). T. denticola cells were counted to quantify their adherent activity. The data presented show a representative result from four independent experiments and are expressed as the means ± SD (n = 30). No significant differences between strains were observed (ANOVA). (TIF) [file pone.0113565.s001.tif]

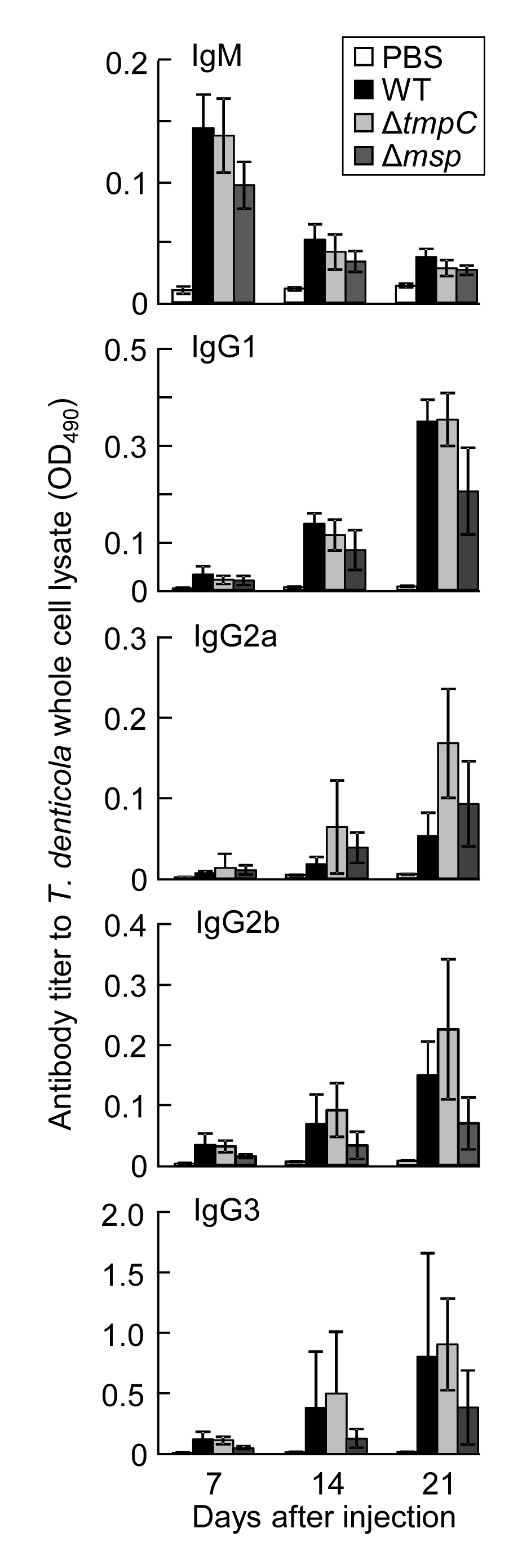

Supplement: Figure S2 — Antibody titer of sera of mice injected with T . denticola strains. Antibody (IgM, IgG1, IgG2a, IgG2b, and IgG3 antibodies) titer of sera of mice injected with T. denticola strains to the whole-cell lysate of the wild type was measured by ELISA. The data are expressed as the means ± SD (n = 6 mice). No significant differences between strains were observed at any point (ANOVA). (TIF) [file pone.0113565.s002.tif]
